# Testicular characterization and spermatogenesis of the hematophagous bat *Diphylla ecaudata*

**DOI:** 10.1371/journal.pone.0226558

**Published:** 2019-12-13

**Authors:** Soraia Fonseca Marinho da Silva, Carlos Henrique de Souza Silva, Fernanda Carolina Ribeiro Dias, Eugenia Cordero-Schmidt, Juan Carlos Vargas-Mena, Ingrid Gracielle Martins da Silva, Sônia Nair Báo, Thaís Gomes de Carvalho, Raimundo Fernandes de Araújo Júnior, Carlos Eduardo Bezerra de Moura, Fabiana Cristina Silveira Alves de Melo, Sérgio Luis Pinto da Matta, Danielle Barbosa Morais

**Affiliations:** 1 Department of Morphology, Federal University of Rio Grande do Norte-UFRN, Natal, Rio Grande do Norte, Brazil; 2 Department of General Biology, Federal University of Viçosa-UFV, Viçosa, Minas Gerais, Brazil; 3 Department of Ecology, Federal University of Rio Grande do Norte-UFRN, Natal, Rio Grande do Norte, Brazil; 4 Department of Cell Biology, University of Brasília-UnB, Brasília, Distrito Federal, Brazil; 5 Department of Animal Sciences, Federal Rural University of the Semi-Arid Region-UFERSA, Mossoró, Rio Grande do Norte, Brazil; 6 Department of Animal Biology, Federal University of Viçosa-UFV, Viçosa, Minas Gerais, Brazil; National Institute of Child Health and Human Development, UNITED STATES

## Abstract

*Diphylla ecaudata* is a hematophagous bat endemic of South America, with food preference for bird blood. Given the lack of information about the reproductive activity of this species, this study aimed to describe the testicular morphology and histomorphometry of *D*. *ecaudata* in order to understand its reproductive biology, specially spermatogenesis. The animals were collected in Lajes city, Rio Grande do Norte, Brazil. Following euthanasia, the testes were histologically processed for morphological, morphometric, ultrastructural and immunohistochemical analyses. Their average body weight was 24.64g, with a gonadosomatic index of 0.49%, tubulesomatic index of 0.47%, and a total of 32.20m of seminiferous tubules per gram of testis. The pre-meiotic, meiotic, and post-meiotic phases accounted for 56.20%, 9.30%, and 34.50% of the seminiferous epithelium cycle, respectively. The ultrastructure of spermiogenesis was similar to that described in other mammals and the perforatorium was not observed in the sperm. Androgen receptors were detected in Sertoli cell nuclei and Leydig cell cytoplasm, while aromatase enzyme was detected only in Sertoli cell nuclei. FGF2 and BCL-2 activities were detected in the cytoplasm of zygotene and pachytene primary spermatocytes, as well as round and elongated spermatids. *D*. *ecaudata* showed testicular pattern similar to other mammals and characteristics common to other bat species. This species stood out for its high efficiency of Sertoli cells, which presented high capacity to support germ cells, besides the highest sperm production rates among those already recorded. This study is the first step towards the knowledge of *D*. *ecaudata* reproduction and the first description of its spermatogenesis.

## Introduction

*Diphylla ecaudata* is a relatively rare species of hematophagous bat. In Rio Grande do Norte state, Brazil, it was first recorded in 2017 [1]. This is the second most captured species of hematophagous bats, following *Desmodus rotundus*. It does not cause major economic and epidemiological impacts, mainly due to its feeding preference for the blood of birds [2, 3, 4]. However, since the availability of wild prey for *D*. *ecaudata* was severely reduced in the Caatinga dry forests, a highly modified biome that has been exposed to anthropic pressures and defaunation, domestic birds became more accessible and abundant prey [5, 6]. This dietary flexibility associated with the scarcity of native birds resulted in the first human blood registration in the diet of this species under natural conditions [7]. Thus, the effect of anthropogenic impacts on the ecological balance of *D*. *ecaudata* also reflects in its medical-sanitary and economic relevance. Therefore, it is important to understand the reproductive biology of the species aiming to maximize rational management actions.

The knowledge on *D*. *ecaudata* gametogenesis is extremely limited, and one factor that contributes to the scarcity of studies on its reproduction is that this is a secretive species which has a more restricted distribution when compared to other bats, especially those with a hematophagous habit [4]. The few studies on *D*. *ecaudata* reproduction are based mainly on ecological and behavioral studies of female. *D*. *ecaudata*, a polygynous species, has a gestation period of approximately 5.5 months, with births occurring during spring and summer, which coincides with the birth of domestic and native birds in Latin America [8]. Usually, only one animal per litter is born and, occasionally, two offspring can be generated per year [9]. No studies were found on the male reproductive activity of this species. Therefore, the present study aimed to describe the morphology of the testes of *D*. *ecaudata*, as well as the testicular histomorphometry, in order to understand its reproductive biology and spermatogenesis. So, this study represents an extra effort to increase understanding of reproductive patterns in bats, specially *D*. *ecaudata*, which could contribute to developing of conservational programs regarding this species, face to the anthropogenic pressures on its natural area.

## Materials and methods

### Study area and animals collection

The animals were collected in Lajes city, Rio Grande do Norte, Brazil (05º42'00"S, 36º14'41"W), in February (n = 1), July (n = 3) and September (n = 2) of 2017. This is a tropical area with a warm and humid weather, without a clear distinction among the seasons of the year [10]. Usually are stablished only the dry season (September to February) and the rainy season (March to August) [11]. The captures were authorized by the Chico Mendes Institute for Biodiversity Conservation (ICMBio, license number 55562–1). All experimental procedures were conducted in accordance with the recommendations of the National Council for Animal Experimentation Control (CONCEA). The protocol was approved by the Ethics Committee on Animal Use of the Federal University of Rio Grande do Norte (CEUA UFRN, protocol number 056/2016). All efforts were made to minimize animal suffering.

Six adult *D*. *ecaudata* males were captured at nightfall using mist nets at the entrance to the abandoned ore galleries, which animals used as shelters. Adult animals were identified based on the fusion of the epiphyseal cartilage of the fourth finger at the metacarpal-phalangeal junction [12].

The animals were transported in bags suitable for containment and transport of bats to Natal city, Rio Grande do Norte, Brazil, and the euthanasia was performed on the same day. The animals were anesthetized intraperitoneally (xylazine 50 mg/kg and ketamine 80 mg/kg), weighed and subsequently euthanized by deepening the anesthetic plane (xylazine 150 mg/kg and ketamine 240 mg/kg).

### Histological processing

One testis of each animal was fixed in Karnovsky solution [13] for 24 hours and histologically processed for either morphological and morphometric analyses under light microscopy, or for ultrastructural analysis, under transmission electron microscopy.

Testicular fragments were embedded in glycol methacrylate (Historesin, Leica), cut into 3-μm sections using a rotatory microtome (Leica RM 2245), and stained with toluidine blue/sodium borate 1% (Merck) for light microscopy analyses. For ultrastructural analysis, testicular fragments were post-fixed with 2% osmium tetroxide and 1.6% potassium ferricyanide in 0.2 M sodium cacodylate buffer, followed by staining in 0.5% aqueous solution of uranyl acetate, overnight. Dehydration was performed in ethanol and acetone, followed by the addition of embedding resin (Spur, Sigma-Aldrich^®^). Ultrathin sections were contrasted with uranyl acetate and lead citrate and observed under a transmission electron microscope (JEOL 1011).

The other testis of each animal was fixed in 4% Paraformaldehyde, processed for embedding in histological paraffin and destined for immunohistochemical analyses. Testicular sections with 4 μm thickness were obtained on signaled slides. The histological sections were deparaffinized, rehydrated, washed in 0.3% Triton X-100 in phosphate buffer and incubated with endogenous peroxidase (3% hydrogen peroxide). The sections were incubated overnight at 4° C in the presence of primary antibodies (Santa Cruz Biotechnology) against pre-apoptotic protein BCL-2 (1: 400), fibroblast growth factor FGF2 (1: 400), aromatase (1: 200), and androgen receptor (1: 200). The sections were carefully rinsed with phosphate buffer and incubated in the presence of secondary antibody streptavidin/HRP-conjugated (Biocare Medical) for 30 minutes. Immunoreactive cells were visualized by colorimetric detection following the protocol provided by the manufacturer (TrekAvidin-HRP Label + Kit Biocare Medical). The sections were counterstained with hematoxylin and the labeled positive areas were captured by a photomicroscope (Nikon E200 LED).

Considering each used antibody, the number of positive cells per tubular cross section was quantified in relation to the number of cells without immunostaining in an area of approximately 40,000 μm^2^. The following formula was used: [(number of marked cells / number of unmarked cells) / number of analyzed sections].

### Testicular morphometry

Both testes were weighted after fixation, using an analytical balance (BEL M214AIH). The gonadosomatic index (GSI) was calculated by dividing the testes weight by body weight and multiplying by 100, in order to quantify the investment in the testicles regarding the total body mass.

Digital images were obtained using a light-field photomicroscope (Olympus BX-50 or BEL Bio2/3 Eurekam 5.0) and analyzed using the Image-Pro Plus^®^ software. Then, the volumetric proportions of all components of the seminiferous tubule (tunica propria, seminiferous epithelium and lumen), intertubule and tunica albuginea were determined after counting 3,520 intersection points, per animal, in 10 square grids randomly placed over these digital images (100x magnification). In order to obtaining their percentages, the counting obtained for each element in each image was divided by the number of points scored, multiplying this value by 100. Seminiferous tubules and intertubule volumes were calculated by multiplying the testes' weight by their respective percentages and dividing these values by 100 [14, 15]. Since the mammalian testis density is around 1 [16], its weight was considered equal to the volume.

The tubulesomatic index (TSI) was calculated in order to quantify the investment in the seminiferous tubules regarding the total body mass. It was obtained by dividing the tubular volume by the body weight and multiplying the result by 100. The mean tubular diameter was obtained by measuring 20 tubular cross-sections per animal, regardless the stage of the cycle. These sections were also used to measure the height of the seminiferous epithelium, from the tunica propria to the tubular lumen, taking two diametrically opposite measurements in each cross section [14, 15].

The seminiferous tubule length (STL, in meters) per testis was estimated as follows: STL = STV/ лR^2^ (STV = seminiferous tubule volume; лR^2^ = tubule area; R = tubular diameter/2). The STL was divided by the testicular weight to calculate the length of the seminiferous tubules per gram of testis (STL/g), to allow comparisons between different species [14, 15].

Coincident points (n = 1000) over the intertubular components were recorded: Leydig cell, blood and lymphatic vessels, and connective tissue. The volumetric rates of these components were also estimated (400x magnification). The percentage of these components in the intertubule was estimated by multiplying the total number of points on each component by 100 and dividing the obtained value by 1000. The percentage of these components in the testis was obtained by multiplying the percentage of intertubule by the percentage of each component in the intertubule and dividing the obtained value by 100. The volume of each intertubular component in the testicular parenchyma was calculated by the following formula: (percentage of each component in the testis x gonadal weight) / 100. The values were expressed in μL [14, 17].

The mean diameter of the Leydig cell was obtained after measuring 30 cells per animal, selecting those with the most spherical nuclei and evident nucleoli. The nuclear volume was obtained by using the formula 4/3 πR^3^, where R = nuclear diameter/2. The cytoplasmic volume was estimated by multiplying the percentage of cytoplasm by the nuclear volume, divided by the nuclear percentage. The single cell volume was estimated by adding the nuclear volume to the cytoplasmatic volume. These values were expressed in μm^3^. The total volume occupied by the Leydig cells in the testicular parenchyma was obtained by multiplying the percentage of Leydig cells in the testis by the gonadal weight and dividing the obtained value by 100. The number of Leydig cells per testis was estimated from the Leydig cell individual volumes and the total volume occupied by these cells in the testicular parenchyma. This value was divided by the gonadal weight to estimate the number of Leydig cells per gram of testis. The Leydigosomatic index (LSI), which quantifies the investment in Leydig cells to body mass, was estimated by dividing the Leydig cell volume in the testicular parenchyma by the body weight and multiplying by 100 [14, 17].

### Stages of the seminiferous epithelium cycle

The stages of the seminiferous epithelium cycle of *D*. *ecaudata* were characterized according to the tubular morphology method [18], based on the shape and position of different germ cells within the epithelium and on the occurrence of meiotic divisions. The relative frequency of each stage described was taken after random characterization and counting of 200 cross sections of seminiferous tubules per animal [14, 19].

### Cell counts and spermatogenic yield

The number of each cell type found at Stage 1 of the seminiferous epithelium cycle was estimated by counting their nuclei (germ cells) or nucleoli (Sertoli cells) in 10 random tubular cross sections per animal. Thirty nuclear diameters of type-A spermatogonia (SPTG A), primary spermatocytes in preleptotene/leptotene (PL/L), primary spermatocytes in pachytene (PC), round spermatids (RS) and Sertoli cells (SC) nuclei were measured for each animal. The results were corrected due to variations in the size of the cells and the section thickness, as described by [20].

The intrinsic yield of spermatogenesis was calculated based on the ratio between corrected germ cell numbers, in order to quantify spermatogenesis efficiency. The mitotic index (PL/L : SPTG A) was calculated to determine the loss or degeneration that occurred during the spermatogonial phase; the meiotic index (RS : PC), so as to determine the efficiency of the meiotic divisions; and the overall yield of spermatogenesis (RS : SPTG A) to quantify the efficiency of the spermatogenic process [14, 19].

The total Sertoli cell support capacity was calculated using the sum of all germ cells types divided by the number of Sertoli cells ((SPG A + PL/L + PC + RS) : SC). The total number of Sertoli cells per testis was obtained by multiplying their corrected number by the tubular length per testis (in μm) and dividing the result by the section thickness [14]. The obtained results were divided by the testicular weight in order to calculate the number of Sertoli cells per gram of testis [14, 19].

The cell loss in spermiogenesis was assumed to be nonsignificant [21] and the spermatic reserve of the testis (SRT) was calculated on the basis of the round spermatid populations, using the formula: SRT = (seminiferous tubule length / cut thickness) × corrected number of round spermatids per cross-section [14, 15, 18].

### Statistical analysis

The results were submitted to descriptive statistical analysis and the averages obtained were expressed as mean ± standard deviation.

## Results

### Biometry and seminiferous tubule morphometry

**Fig 1:**
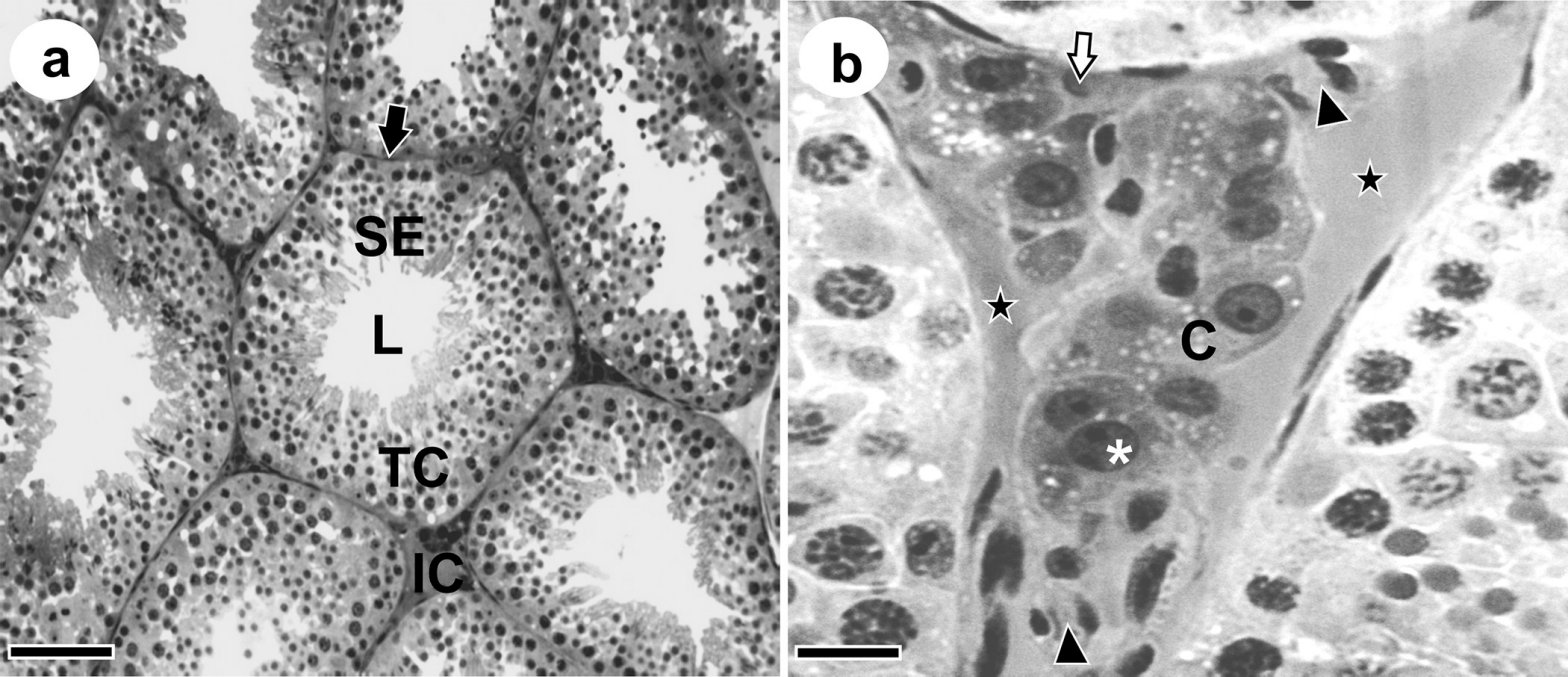
Cross sections of *Diphylla ecaudata* testis. TC: Tubular Compartment; SE: Seminiferous Epithelium; L: Lumen; Black arrow: tunica propria; IC: Intertubular Compartment; *: Leydig cell nucleus; C: Leydig cell cytoplasm; White arrow: Connective tissue; 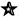: Lymphatic vessel; ►: Blood vessel. Scale Bars: a: 30 μm, b: 10 μm.

Table 1 contains the mean values for biometry and volumetric proportions of testicular parenchyma components of *D*. *ecaudata*, as presented in Fig 1. The testicular parenchyma was predominantly composed of seminiferous tubules, and the tubular compartment, mainly of seminiferous epithelium.

**Table 1:** Biometry and morphometry of the testicular components of *Diphylla ecaudata*. The data are reported as mean ± standard deviation (SD) of the mean.

Parameters	Mean ± SD (n = 6)
Body weight (g)	24.64 ± 1.45
Testes weight (g)	0.12 ± 0.04
Gonadosomatic Index (%)	0.49 ± 0.17
Tunica albuginea (%)	8.39 ± 1.27
Seminiferous tubules (%)	95.98 ± 0.92
Tunica propria (%)	4.49 ± 0.61
Epithelium (%)	63.37 ± 3.13
Lumen (%)	28.13 ± 3.44
Intertubule (%)	4.02 ± 0.92
Seminiferous tubules volume (mL)	0.12 ± 0.04
Tubulesomatic Index (%)	0.47 ± 0.16
Tubular diameter (μm)	195.09 ± 6.00
Epithelium height (μm)	44.26 ± 4.31
Seminiferous Tubules Length per testis (m)	3.90 ± 1.31
Seminiferous Tubules Length per gram of testis (m/g)	32.20 ± 2.00

### Intertubular morphology and morphometry

Table 2 presents the histomorphometry of the intertubular compartment of *D*. *ecaudata*. This compartment was predominantly composed of Leydig cells, followed by blood vessels, lymphatic vessels and connective tissue (Fig 1B). The occupation of intertubule and testicular parenchyma by blood vessels was similar to that of lymphatic vessels, while the volume of lymphatic vessels by testicular parenchyma was greater than the volume of blood vessels. The Leydig cell morphometry is presented in Table 3.

**Table 2:** Volumetric proportion (%) and volume of the intertubular compartment of *Diphylla ecaudata*. The data are reported as mean ± standard deviation (SD) of the mean.

Parameters	Mean ± SD (n = 6)
Intertubule volume (mL)	0.006±0.002
Percentage in the intertubule (%)	
Leydig cells	48.45±14.31
Blood vessels	24.20±9.06
Lymphatic vessels	20.27±19.12
Connective tissue	7.08±3.25
Percentage in the testicular parenchyma (%)	
Leydig cells	2.26±1.10
Blood vessels	1.12±0.61
Lymphatic vessels	0.97±0.94
Connective tissue	0.34±0.18
Volume per testicular parenchyma (μL)	
Leydig cells	2.52±0.86
Blood vessels	1.23±0.38
Lymphatic vessels	1.42±1.67
Connective tissue	0.44±0.29

**Table 3:** Morphometry of the Leydig cell of *Diphylla ecaudata*. The data are reported as mean ± standard deviation (SD) of the mean.

Parameters	Mean ± SD (n = 6)
Nuclear diameter (μm)	13.32±2.31
Nuclear percentage (%)	26.97±7.41
Nuclear volume (μm^3^)	1335.73±758.16
Cytoplasmic percentage (%)	73.03±7.41
Cytoplasmic volume (μm^3^)	4305.02±3744.37
Leydic cell volume (μm^3^)	5640.75±4469.36
Number of Leydig cells per testis (x10^5^)	5.78±3.05
Number of Leydig cells per gram of testis (x10^5^)	47.82±16.64
Leydigosomatic index (%)	0.005±0.006

### Stages of the seminiferous epithelium cycle (SEC)

**Fig 2:**
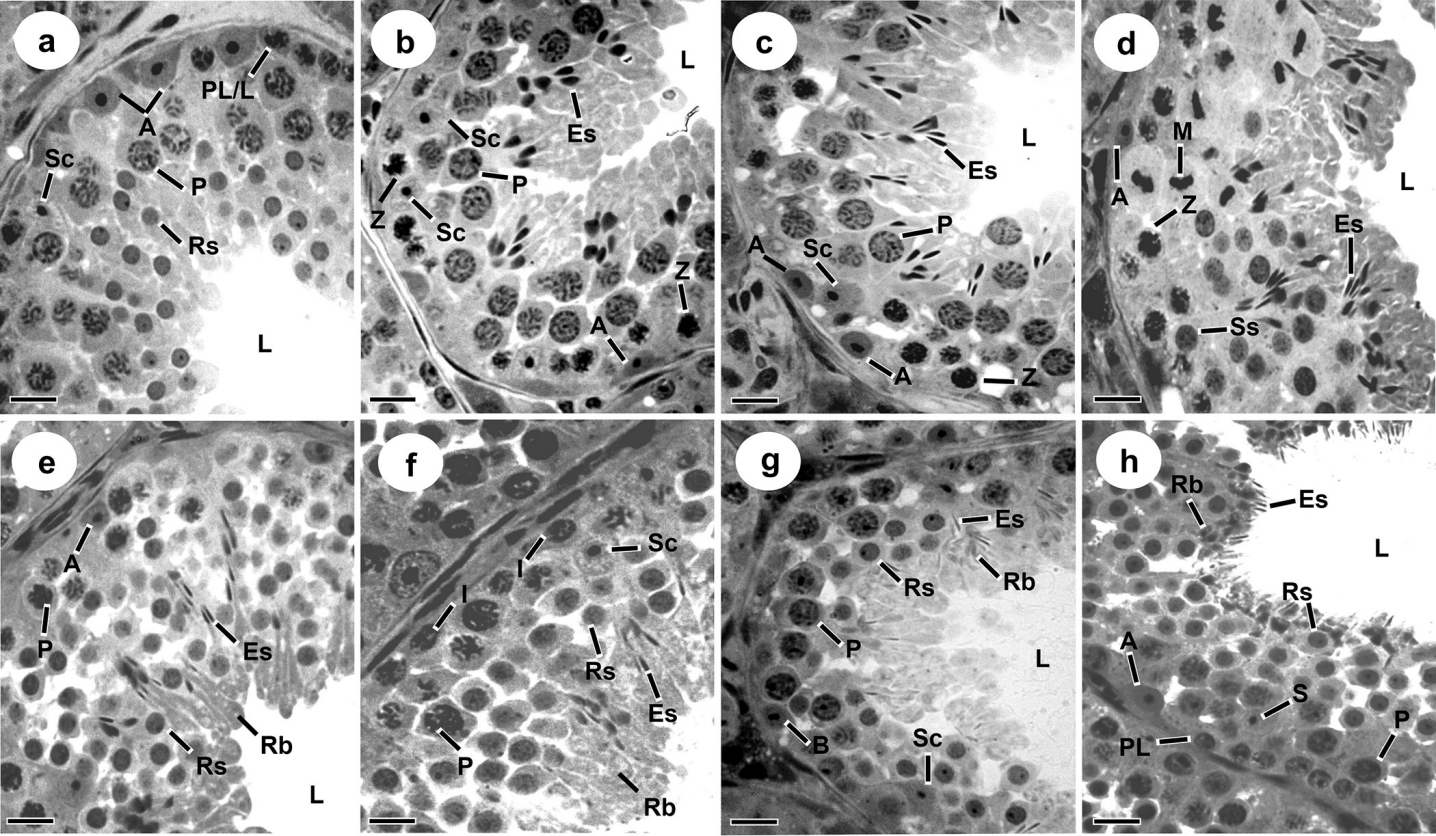
Stages of the seminiferous epithelium cycle (SEC) of *Diphylla ecaudata* according to the Tubular Morphology Method. a: Stage I; b: Stage II; c: Stage III; d: Stage IV; e: Stage V; f: Stage VI; g: Stage VII; h: Stage VIII. Sc: Sertoli cell nuclei; A: Type A spermatogonia; I: Intermediate spermatogonia; B: Type B spermatogonia; PL/L: Preleptotene/Leptotene primary spermatocyte; Z: Zygotene primary spermatocyte; P: Pachytene primary spermatocyte; M: Meiotic division; Ss: Secondary spermatocyte; Rs: Round spermatid; Es: Elongated spermatid; Rb: Residual body; L: Lumen. Scale bar: 20 μm.

Fig 2 shows the SEC of *D*. *ecaudata*, which is divided into eight stages, as described by Berndston (1977). Sertoli cells (SC) and type-A spermatogonia (SPG A) were found in all stages. During spermatogonial mitosis, type-A spermatogonia goes through transition to the intermediate type, which was found at stage 6, while type-B spermatogonia was found at stage 7. The type B spermatogonia originates the primary spermatocyte in preleptotene at stage 8 and this cell begins the first meiotic division. The transition from preleptotene to leptotene occurs at stages 1 to 2, originating zygotene primary spermatocyte at stage 2. This spermatocyte was observed until stage 4, which originated the pachytene primary spermatocyte. In this stage, the pachytene spermatocyte originated the diplotene primary spermatocyte, thus finishing the first meiotic division, followed by the second meiotic division and originating the secondary spermatocytes. Since the second meiotic division is faster than the first, the secondary spermatocyte quickly originated the round spermatids still at stage 4. The round spermatid begins the elongation process only at stage 2 and can be found until the end of the current SEC, at the beginning of the next cycle. Thus, elongated spermatids emerge from stage 3 and can be viewed up to stage 8.

**Fig 3:**
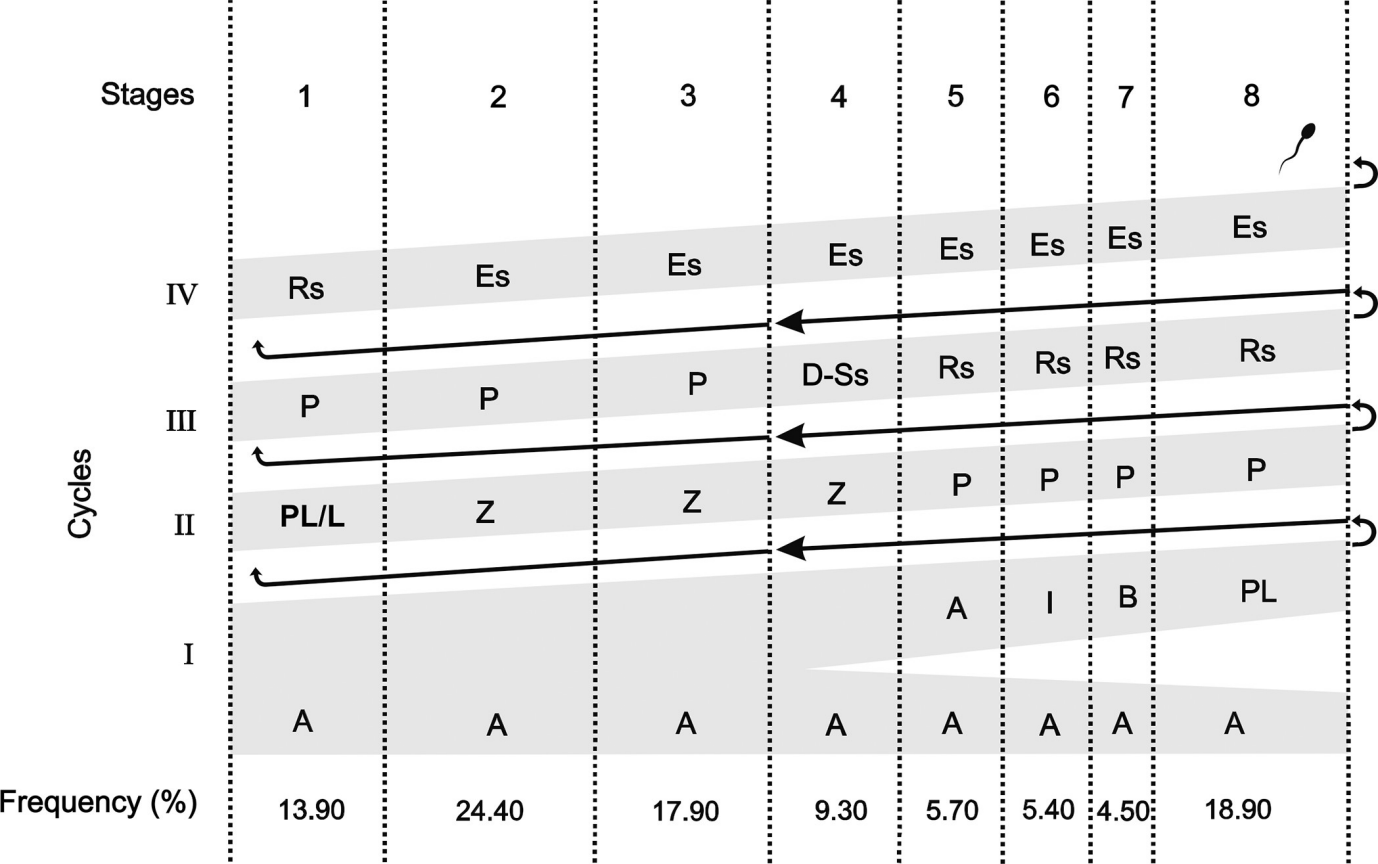
Diagram of the spermatogenic process of *Diphylla ecaudata* representing the progression of the germ cells alongside the stages of the seminiferous epithelium cycle and the frequency (%) of each stage. Each row corresponds to a generation of spermatogenic cells and each column corresponds to a stage. Roman numbers indicate the cycles of cell division necessary to complete the spermatogenesis process. A: Type A spermatogonia; I: Intermediate spermatogonia; B: Type B spermatogonia; PL/L: Preleptotene/Leptotene primary spermatocyte; Z: Zygotene primary spermatocyte; P: Pachytene primary spermatocyte; D: Diplotene primary spermatocyte; Ss: Secondary spermatocyte; Rs: Round spermatid; Es: Elongated spermatid.

While different spermatocyte generations can be seen at stages 1 to 8, only one spermatid generation is observed at stages 1 to 3 and two generations at stages 5 to 8 (Figs 2 and 3). Stage 4 is characterized by diplotene primary spermatocyte division to originate secondary spermatocytes, which divide to produce round spermatids. The round spermatids begin elongation at stage 2 and will reach the lumen at stage 8.

**Fig 4:**
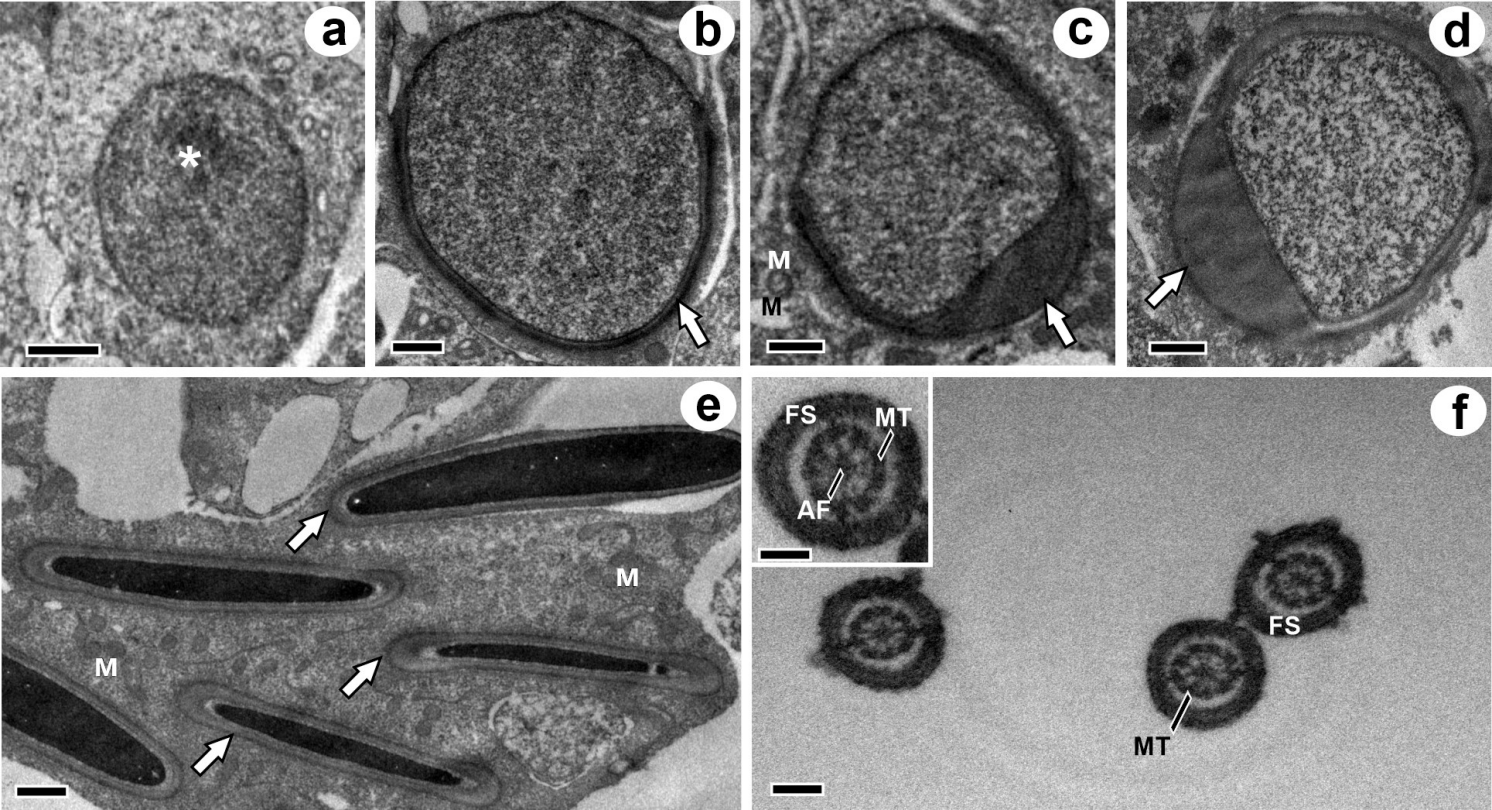
Ultrastructural aspects of spermiogenesis of *Diphylla ecaudata*. a: round spermatid without acrosomal cap, at stage 5 of seminiferous epithelium cycle; b-d: spermatid elongation with different degrees of nuclear association of the acrosomal cap; e: elongated spermatids with complete formation of the acrosome cap; f: transverse sections of the flagellum. *: nucleoli; White arrow: acrosome; M: mitochondria; FS: fibrous sheath; AF: axial filament; MT: microtubule. Scale bars: a: 2 μm, b: 0.5 μm, c: 1 μm, d: 0.2 μm, e: 0.5 μm, f: 0.2 μm, detail: 0.2 μm.

The process of spermatid elongation involves progressive reduction of the cytoplasmic area concomitant with nuclear flattening, association of the acrosomal cap to the nuclear surface and development of the sperm tail. In this sperm region, the cytoskeleton had an axial filament composed of a central pair of microtubules surrounded by the fibrous sheath composed of 9 pairs of peripheral microtubules. No perforatorium occurrence was detected (Fig 4).

According to the frequency of each SEC stage in *D*. *acaudata* (Fig 3), the pre-meiotic (stages 1 to 3), meiotic (stage 4), and post-meiotic (stages 5 to 8) phases account for 56.20%, 9.30%, and 34.50%, respectively.

### Cell counts and spermatogenic yield

The corrected numbers of germ and Sertoli cells at stage 1 of SEC are described in Table 4. The population of preleptotene/leptotene and pachytene primary spermatocytes were similar. Considering all the cells that composed the seminiferous epithelium in *D*. *ecaudata* at stage 1, each Sertoli cell was able to support on average 30 germ cells.

**Table 4:** Corrected number of germ and Sertoli cells per tubule cross section at stage 1 of the seminiferous epithelium cycle (SEC) and spermatogenic indexes of *Diphylla ecaudata*. The data are reported as mean ± standard deviation (SD) of the mean.

Parameters	Mean ± SD (n = 6)
Sertoli cells	2.83±0.63
Type A spermatogonia	0.90±0.40
Preleptotene/leptotene primary spermatocytes	15.00±4.24
Pachytene primary spermatocytes	17.67±3.39
Round spermatids	49.33±10.51
Mitotic index	19.37±11.13
Meiotic index	2.81±0.38
Spermatogenic yield	67.03±41.19
Sertoli cell support capacity	30.16±7.26
Sertoli cell/testis (x10^5^)	37.67±14.17
Sertoli cell/g of testis (x10^6^)	59.44±13.36
Spermatic reserve/testis (x10^6^)	69.36±40.06
Spermatic reserve/g of testis (x10^7^)	103.65±21.67

### Immunohistochemical analysis

**Fig 5:**
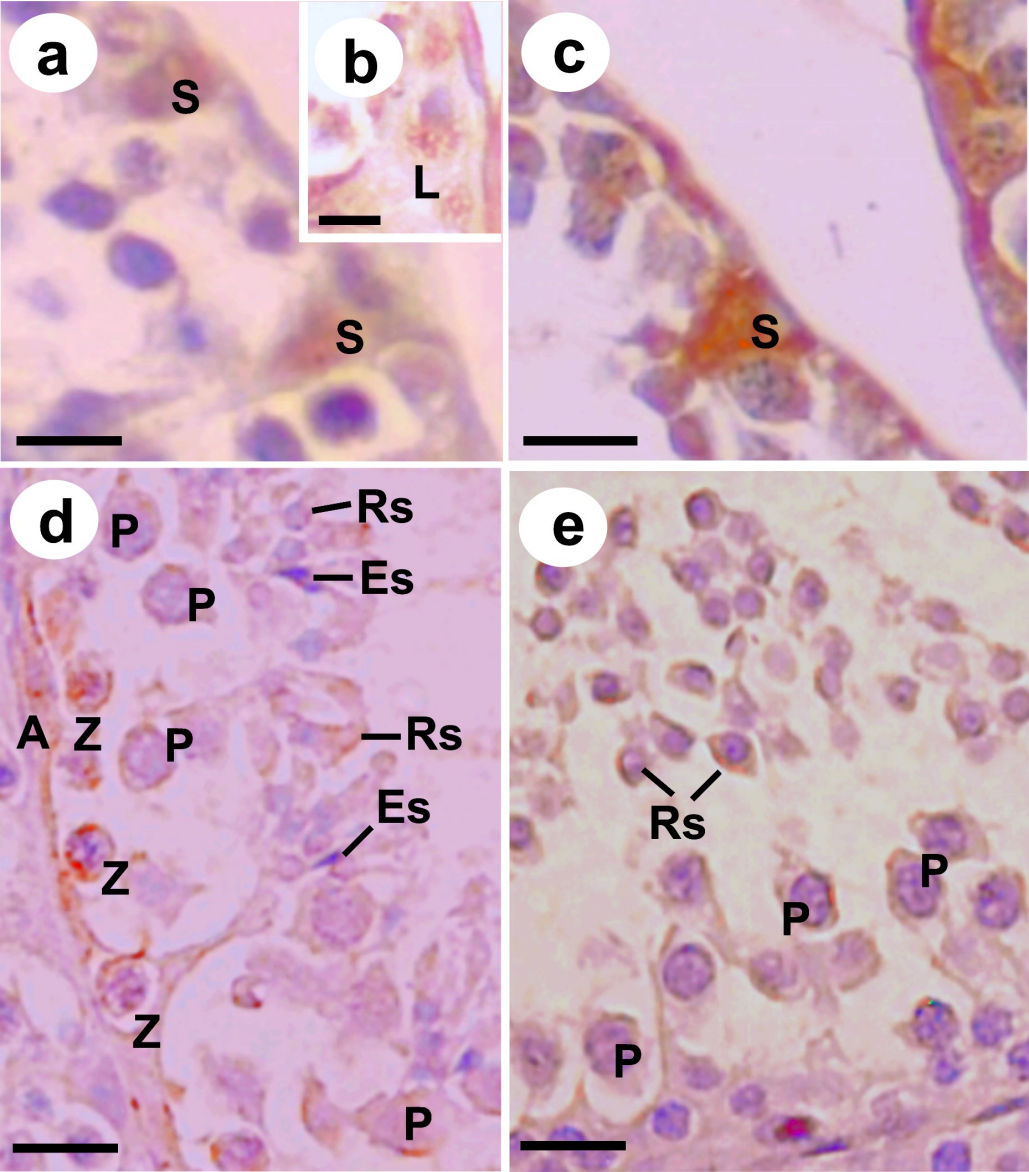
Immunohistochemical detection of androgen (a-b), aromatase (c), FGF2 (d) and BCL-2 (e) receptors on *Diphylla ecaudata* testes. S: Sertoli cell nuclei; L: Leydig cell cytoplasm; A: Type A spermatogonia; Z: zygotene primary spermatocyte; P: pachytene primary spermatocyte; Rs: round spermatid; Es: elongated spermatid. Scale bars: 30 μm.

Androgen receptors showed immunostaining in Sertoli cell nuclei (Fig 5A) and Leydig cell cytoplasm (Fig 5B), while aromatase enzyme was detected only in Sertoli cell nuclei (Fig 5C). Immunostaining for FGF2 (Fig 5D) and BCL-2 (Fig 5E) was detected in the zygotene and pachytene primary spermatocytes, besides round spermatids (Table 5). The elongated spermatids showed a discrete immunoreactivity, and only for FGF2 (Fig 5D).

**Table 5:** Expression frequency of the androgen receptor, aromatase, FGF 2 and BCL-2 of *Diphylla ecaudata* testes. The data are reported as mean ± standard deviation (SD) of the mean.

Cell type	Androgen	Aromatase	FGF 2	BCL-2
Sertoli cell	0.32 ± 0.18	1.00 ± 0.09	---	---
Leydig cell	0.02 ± 0.04	---	---	---
Zygotene primary spermatocyte	---	---	1.00±0.79	1.00±1.00
Pachytene primary spermatocyte	---	---	1.00±0.12	1.00±0.16
Round spermatid	---	---	1.00±0.17	1.00±0.00
Elongated spermatid	---	---	1.00±0.31	---

## Discussion

This study stands out for being the first to describe the spermatogenic process of the hairy-legged vampire bat *D*. *ecaudata*. The few studies on the reproduction of this species are based mainly on behavioral aspects related to the females, while no studies on the gametogenesis of males were found.

The difficulty in collecting these animals must be considered, as they have the most restricted distribution among vampire bats, fast moving and dislocate quickly to other shelters when disturbed [4]. So, due to the limited sample size, it wasn’t possible to infer on this study about reproductive seasonality, and this study focuses on the testicular morphology and spermatogenic yields of this species.

### Biometry and seminiferous tubule morphometry

*Diphylla ecaudata* is the smallest species of hematophagous bat [4], weighing approximately 25 g. The two other known hematophagous bat species, *Desmodus rotundus* and *Diaemus youngi*, have an approximate body weight of 36–42 g [8, 14] and 30–38 g [22], respectively. The gonadal weight of *D*. *ecaudata* was similar to that previously reported for other bat species [15, 23, 24].

The GSI and TSI found for *D*. *ecaudata* were also similar to those observed in other neotropical bat species [14, 15, 24, 25]. As most of these species live in harem systems, consisting of a dominant male and groups of 8 to 12 adult females [8, 26], it is required a greater investment in gonads when compared to monogamous animals, as observed in several mammals [27, 28]. In bats, it was observed that species in which multiple males roost with multiple females shows the largest relative testes, single-male/multi-female species are intermediate in testes size, and the smallest relative testes occurs in single-male/single-female species [28, 29]. So, despite the association between testes size and mating systems in bats are multifactorial, the GSI and TSI for *D*. *ecaudata* reinforce the literature data regarding a polygynic mating system on this species, as well in other bats [15, 26].

The arrangement of testicular parenchyma and its percentages, with seminiferous tubules and intertubule, were similar to those reported in other bats, such as *D*. *rotundus* [14]. However, these phylostomids of the subfamily Desmodontinae present a slightly higher percentage of testicular parenchyma than other individuals of the families Phyllostomidae and Molossidae [15, 24]. The percentage of tubular compartment represented by seminiferous epithelium in *D*. *ecaudata* was lower than that recorded for other bat species [14, 15, 24].

The tubular diameter (195.09 μm) was higher than that observed in other bats (137.50 μm [30]; 139.54 μm [24]; 175.00 μm [31]; 188.04μm [14]), which is close to the value presented by the frugivorous bat *Artibeus lituratus* (200.7 μm [23]). The height of the seminiferous epithelium was within the range observed in these animals. The mean tubular length per gram of testis was lower than that recorded for other bats (48.91 to 79.63 m, [15, 24]), but close to that reported for *D*. *rotundus*, (34.70 m [14]).

### Intertubular morphology and morphometry

The intertubular compartment of *D*. *ecaudata* seemed morphologically similar to that described for other mammals, consisting of Leydig cells, blood and lymphatic vessels, and connective tissue. However, its percentage within the testicular parenchyma was the lowest recorded [17, 32, 33], which was similar to that found in the hematophagous bat *D*. *rotundus* [14].

Leydig cells were the main component of *D*. *ecaudata* intertubular compartment, which is also observed in other bat species [14, 17, 33]. The largest investment in these cells is directly related to the matting pattern of this species and its polygynic behavior [8]. Therefore, they require greater androgenic investment when compared with monogamous species, such as the crab-eating fox [34]. The number of Leydig cells per gram of testis found in *D*. *ecaudata* (47.82 x 10^5^ cells) was lower than the observed for frugivorous bat *S*. *lilium* (11.3 x 10^7^ cells) [17] and the insectivorous bat *M*. *molossus* (48.49 x 10^6^ cells) [33].

The Leydigosomatic index for *D*. *ecaudata* (0.005%) was smaller to that observed in other bat species, whose average ranged from 0.015% to 0.04% [14, 35]. This index was close to that found in other mammals, such as mice and ocelots, 0.007% and 0.0036% respectively [36, 37]. Both the nuclear diameter of Leydig cells and their volumes were larger than those found for other bat species and other mammals [14, 17, 33, 38, 39]. This higher investment in Leydig cell nuclear diameter and volume compared to its number suggest an alternative to guarantee the concentration of testosterone to maintain the libido and ensure the protection of the harem.

### Stages of the seminiferous epithelium cycle (SEC)

In *D*. *ecautada*, as well as in other bats and mammals, the SEC is divided into eight stages, as described by Berndston [18]. Stage 2 was the most frequent, while in other species, stage 1 is usually the most observed [14, 15, 19, 33, 35, 40, 41]. Zygotene primary spermatocytes emerged only from stage 2, similarly to the observed in most mammals already studied. However, it differs from the observed in other bat species, such as insectivore *Molossus molossus* and frugivore *Sturnira lilium*, in which these cells were found at stage 1 [15, 19, 32, 42]. The pachytene primary spermatocyte is found at all stages, since this phase may last for hours, days or even weeks, depending on the species [14, 19, 38, 39, 41].

Spermatogonia are present in all SEC stages due to their constant mitotic activity. Thus, type A spermatogonia could be observed in all stages, as well as intermediate type at stage 6 and type B at stage 7, as reported in *M*. *molossus*, *S*. *lilium*, *D*. *rotundus* and *Myotis levis* bats [14, 19, 43] and other mammals, such as domestic cat and mice [36, 38]. In guinea pig, however, intermediate spermatogonia were observed at stage 5, and type B spermatogonia, at stages 6 and 7 [39].

The ultrastructural analysis of spermatids showed the acrosome formation caused by the agglutination of the Golgi complex pro-acrosomal vesicles and adhesion to the nuclear surface, which occupies about two-thirds of the nucleus in mature mammalian sperm [44, 45]. No perforatorium was observed in the present study. This structure is related to sperm penetration into the oocyte cytoplasm and is poorly developed or absent in several bat families [46–48]. The early stages of flagella formation were also evident in the region that will originate the sperm tail. The axial filament and microtubules were observed in the tail end portion, and the organization of the axial filament was lost along the length of the end piece. This pattern of microtubule organization showed by *D*. *ecaudata* was similar to that found in other bat species [49–52].

### Cell counts and spermatogenic yield

While the germinative cell population at stage 1 of the SEC in *D*. *ecaudata* was similar to that observed in other bat species, the amount of Sertoli cells was considerably smaller, which reflected in a smaller number of these cells per gram of testis. Thus, while *D*. *ecaudata* showed approximately 2.8 Sertoli cells per tubular cross-section at stage 1 and 59 x 10^6^ Sertoli cells per gram of testis, *D*. *rotundus*, *M*. *molossus* and *S*. *lilium* presented, respectively, 5.76, 8.48 and 8.51 Sertoli cells at stage 1 and 13.10 x 10^7^, 28.09 x 10^7^ and 22.31 x 10^13^ Sertoli cells per gram of testis [14, 15, 24]. On the other hand, the support capacity of Sertoli cells was approximately 30 cells, which is higher than that observed in other mammals (range from 10 to 22 cells) [32, 42, 39, 53– 55] and indicates the higher efficiency of these cells in *D*. *ecaudata*.

The mitotic index of *D*. *ecaudata* (19.37%) was higher than that observed in *D*. *rotundus* (16.93% [14]), *S*. *lilium* (15.48% [24]) and *M*. *molossus* (13.76% [15]), while the meiotic index and the spermatogenic yield were similar between these bat species. The sperm reserve per gram of testis of *D*. *eucadata* (103.65 x 10^7^ cells) was considerably higher than that found in *S*. *lilium* and *M*. *molossus* (range from 56.64 x 10^7^ to 76.52 x 10^7^ cells) and in other mammals (range from 103.80 x 10^6^ to 165.90 x 10^6^ cells) [56, 57]. This index is calculated based on the seminiferous tubule length and the round spermatid population, since cell loss during spermiogenesis is considered nonsignificant [21]. Thus, the round spermatid population is considered a safe parameter to determine the number of sperm produced [32]. This finding indicates that *D*. *ecaudata* presents the highest sperm production rates among those already recorded.

### Immunohistochemical analysis

This is the first study describing the expression of androgen receptors, aromatase, FGF2 and BCL-2 in *D*. *ecaudata* testes, which provides knowledge about the cells responsive to these important factors related to the spermatogenesis regulation.

*D*. *ecaudata* expressed androgen receptors more often in Sertoli cells than in Leydig cells. Similarly, these receptors also showed more discrete expression in Leydig cells of *A*. *lituratus*, which indicates that this cell population is more regulated by estrogen than androgen [58]. Aromatase expression has been detected in Leydig cells, Sertoli cells, spermatocytes, spermatids and sperm from mice, rats, sheep and horses [59–63]. However, in *D*. *ecaudata* its expression was observed only in Sertoli cells, while in the *Myotis nigricans* bat its expression was observed in elongated spermatids, Sertoli and Leydig cells [64].

Fibroblast growth factors (FGFs) are polypeptides that act on cell proliferation, meiosis and cell differentiation [65]. The FGF2 expression in *D*. *ecaudata* was observed in zygotene and pachytene primary spermatocytes, as well as in round and elongated spermatids. However, in other mammals, such as rodents, deer, cattle and humans, FGF2 was detected exclusively in Leydig cells and spermatogonia [66–69]. The expression of the anti-apoptotic protein BCL-2 was similar to that presented by the rodent *Lagostomus maximus*, located in pachytene and zygotene primary spermatocytes and round spermatids [70], which may be related to the common occurrence of these cell types during the SEC, and to the maintenance of the epithelium integrity throughout the cycle.

## Conclusions

The main differences founded in the spermatogenic process of *D*. *ecaudata* were the lower percentage of tubular compartment represented by seminiferous epithelium and the lower tubular length per gram of testis, when compared to other bats and other mammals. On the other hand, differently to that found in other bats, the primary spermatocyte in zygotene emerged only from stage 2 of the seminiferous epithelium and the amount of Sertoli cells was considerably smaller in *D*. *ecaudata*, contrasting with a higher support capacity by these cells, and a higher sperm reserve per gram of testis.

Therefore, *D*. *ecaudata* showed testicular pattern similar to that of other mammals and characteristics common to other bat species, such as large investment in seminiferous tubules and Leydig cells. Although it was expected that the testicular pattern was similar to that found in other bat species, this species stood out for its high efficiency of Sertoli cells, which presented high capacity to support germ cells, and a high spermatic reserve of the testis. The description of the *D*. *ecaudata* spermatogenic process is the first step to obtain knowledge of the male’s reproduction. This information may be useful to correlate with female reproduction and elaborate conservation plans to improve management and prevent the extinction of the species.
